# Progressive Disordered Movements in an Infant Leads to Rare Diagnosis

**DOI:** 10.5811/cpcem.2016.12.32681

**Published:** 2017-01-24

**Authors:** Sarah Pasquale, Aaron Dam, Christopher Kelly, Romaine Schubert, Laura Melville

**Affiliations:** * New York Methodist Hospital, Department of Emergency Medicine, Brooklyn, New York; † New York Methodist Hospital, Department of Neurology, Brooklyn, New York

## Abstract

Desmoplastic infantile ganglioglioma (DIG) is a supratentorial superficially-located cystic neuroepithelial tumor. It is an exceedingly rare tumor with an incidence of <0.1% of central nervous tumors; approximately 60 cases have been reported in the literature. We present a case of a three-month-old infant with progressive disordered movements described as intermittent upper body stiffening with associated eye blinking, drooling, and change in level of alertness. A seizure was witnessed in the emergency department, after which the child was sent for imaging studies. Magnetic resonance imaging (MRI) revealed a large solid and cystic mass in the temporal region measuring 8.6cm × 7.9cm × 5.1cm. The infant underwent complete surgical resection, and post-surgical pathology revealed a diagnosis of DIG. The patient had an excellent post-operative course in the months following discharge. At his last well-child visit, no neurological deficits were appreciated and the infant was meeting expected milestones for his age.

## INTRODUCTION

Desmoplastic infantile ganglioglioma (DIG) is a supratentorial superficially located cystic neuroepithelial tumor. It is an exceedingly rare tumor with an incidence of <0.1% of central nervous tumors, and there have been approximately 60 cases described in the literature.^1^ It commonly presents with increasing head circumference, signs of hydrocephalus, setting sun sign (upward gaze paresis), or, less commonly, seizure activity.^2^ Less common presentations include repeated vomiting, developmental delay, and oculomotor defects; however, sensory and motor deficits are rare.^2^ Here we describe a case of a three-month-old male infant who presented to the emergency department (ED) with a history of “jerking” movements and stiffening of the upper body, worsening over the course of a few weeks. ED work-up revealed a large intracranial mass. Subsequent inpatient evaluation and surgery showed the mass to be a DIG.

## CASE REPORT

A three-month-old full term male was brought to the pediatric ED by his grandmother for evaluation of “jerking” activity worsening in frequency and severity for approximately three weeks. The grandmother described the “jerking” activity as intermittent upper body stiffening with associated eye blinking and drooling with a change in level of alertness just following the event. She reported that the episodes had increased in frequency over the few days prior to ED arrival but claimed the episodes had been present for the approximately one month. The grandmother stated that initially the jerking occurred approximately once per day; however, there were three episodes noted on the day of presentation. She described these episodes as lasting seconds to minutes and were not associated with color change, respiratory distress, or spitting up. They occurred at any time of the day, with no predilection for early morning or sleep arousal, and there was no association with feeding. She denied any recent history of falls, head trauma, fever, cough, congestion, diarrhea or rash. The remainder of the review of systems was negative. The infant was formula-fed every four hours and had been producing a normal amount of wet diapers and stool. When questioned about the pregnancy, the grandmother was unsure if the mother had been using drugs or alcohol, but stated that the infant was the product of an uncomplicated labor and delivery with a normal newborn screening. His birth weight was reported as 2.86 kg. The infant attended daycare, but there were no known sick contacts. The grandmother stated that the baby had been healthy, meeting his development milestones, and gaining weight appropriately according to his last well-child visit. There was a maternal history of multiple sclerosis reported by the grandmother.

On physical exam, the infant appeared well and was noted to make eye contact with the grandmother. Initial weight and vital signs reported at triage were as follows: weight: 5.51 kg, temperature 98.0° F, respiratory rate 22 breaths/min, heart rate 143 beats/min, oxygen saturation of 97% on room air. The pertinent positives of his exam included a full anterior fontanelle but no bulging. Extraocular movements were equal with no nystagmus appreciated. There was a mild left ocular prominence noted but no proptosis. He had no facial deformity or external signs of trauma with tears present and moist mucous membranes. His neck was supple, and appropriate head control was appreciated when he was seated in his grandmother’s lap. An overall slight increase in his tone was noted. All movements were observed to be symmetrical with no obvious motor deficit or weakness.

Moments after the initial assessment, the ED staff was called to the bedside by the grandmother. The infant was observed to have left eye blinking followed by asynchronous right eye blinking with drooling at the mouth. He was unresponsive to direct confrontation. No other jerking motions were observed. The episode lasted less than two minutes during which time he was placed on a cardiac monitor and vital signs were stable. Immediately following the episode, the infant was not responding normally to the grandmother but became increasingly more alert and returned back to baseline within several minutes.

Given his initial presentation and witnessed seizure activity, laboratory tests and imaging were ordered. The results of his lab work were as follows: WBC 10.4 × 10^3^, Hgb 12.4 g/dL, Hct 36.3%, platelets 302 × 10^3^/mm;^3^ Differential: lymphocytes 77%, neutrophils 14%, eosinophils 9%. Sodium 136 mEq/L, potassium 5.5 mEq/L, chloride 106 mEq/L, bicarbonate 20 mEq/L, BUN 5 mg/dL, creatinine 0.22 mg/dL, glucose 100 mg/dL, magnesium 2.1 mEq/L, phosphorus 5.5 mg/dL, anion gap 10.

In an attempt to spare exposure to ionizing radiation, the decision was made to perform a stat non-contrast magnetic resonance imaging (MRI) of the brain. After review of the scout images from MRI, a magnetic resonance angiogram protocol was added. Imaging revealed a large supratentorial cystic and solid mass in the left temporal region measuring 8.6cm × 7.9cm × 5.1cm. A plan was then made to transfer the patient to a pediatric intensive care unit, which could afford a higher level of care. A loading dose of levetiracetam (55 mg) intravenously was administered and D10 NS was run at maintenance.

The infant had five seizures on the day of admission, two of them witnessed. He was given an additional dose of levetiracetam 20 mg/kg IV and then continued on a maintenance dose of 10 mg/kg. The patient was also started on a dexamethasone 0.5 mg/kg. No subsequent seizure activity was reported following medication titration. Operative management was conducted by pediatric neurosurgery on hospital day 6. The patient underwent a left craniotomy with gross tumor resection. Pathology report indicated the tumor was consistent with DIG. In the post-operative period, the patient was observed to have a left lateral gaze palsy, and repeat imaging revealed a new acute infarct of the right thalamus. Due to the acute infarct, a neurology consult was obtained and the infant underwent a full work-up for a new-onset hypercoaguable state. The infant was found to have a slightly elevated anti-thrombin III level, and the infarct was not attributed to surgical management. Echocardiography was found to be normal and the patient was subsequently started on aspirin. The infant’s subgaleal Jackson-Pratt drain remained in place until post-operative day 3. No respiratory or gastrointestinal complications arose and the patient was tolerating full feeds within several days post-operatively.

Despite the right thalamic infarct, the patient had an excellent post-operative course in the months following discharge. At his last well-child visit, no neurological deficits were appreciated and the infant was meeting expected milestones for his age.

## DISCUSSION

DIG is a supratentorial superficially-located cystic neuroepithelial tumor. It is characterized by prominent desmoplasia with neoplastic glial component or neoplastic glioneuronal component. 1, 2 It is an exceedingly rare tumor with an incidence of <0.1% of central nervous tumors. Approximately 60 cases have been reported in the literature. It primarily occurs within the first 24 months with a slight predilection for males over females (1.7:1). 1 It is a benign tumor recognized by the World Health Organization (WHO) under the category of glio-neuronal tumors and is classified by the WHO as a grade I tumor. 3, 4

Clinical presentation is usually consistent with increasing head circumference, signs of hydrocephalus, setting sun sign or, less commonly, seizure activity. Approximately one in four children are diagnosed following a seizure. 2 Less common presentations include repeated vomiting, developmental delay, and oculomotor defects; however, sensory and motor deficits are rare. 2

The supratentorial region is preferentially involved, especially the frontal and parietal lobes, followed by the temporal lobe. 1, 3 The large size of these tumors and the relative lack of obtundation in these children suggest that they are very slow growing and likely congenital in origin.^5^ MRI is the most useful diagnostic tool. Typical imaging reveals a large supratentorial tumor often involving more than one lobe, with both large cystic and solid components with firm attachment to the dura. 2 A recent study suggested that diffusion-weighted imaging may be especially helpful in differentiating DIG from other tumors such as infantile glioblastoma multiforme. 6 Contrast enhancement was reported to be homogenous and avid in DIG tumors, whereas infantile glioblastoma tumors had more heterogeneous uptake. 6 Radical surgical resection is the treatment of choice in DIG. Overall, there is a very good prognosis with complete resection. 1, 2, 5 However, because the mass may be firmly affixed to the dura or may penetrate into deeper cortical layers of the brain this may sometimes be difficult to accomplish, in which case adjunctive chemotherapy or radiation therapy may be used. 2, 6

## CONCLUSION

The differential diagnosis in a three-month-old infant presenting to the ED with new onset seizures is broad. Several important considerations include child abuse, trauma, mass lesions, inborn errors of metabolism, electrolyte disturbance including hypoglycemia and sodium derangements, subtle seizures, jitteriness, infantile spasms (West syndrome), benign myoclonic jerks, and migrating partial epilepsy in infancy. Febrile seizures are only considered in infants greater than six months of age. If fever does occur with seizure in a child younger than this, it is a harbinger of a more serious underlying etiology. Our case was a three-month-old infant with worsening intermittent generalized seizures of unknown origin. MRI revealed a large, superficially located solid and cystic mass in the left parietal lobe. Pathology of the tumor following total resection revealed the diagnosis of desmoplastic infantile ganglioglioma.

## Figures and Tables

**Image:**
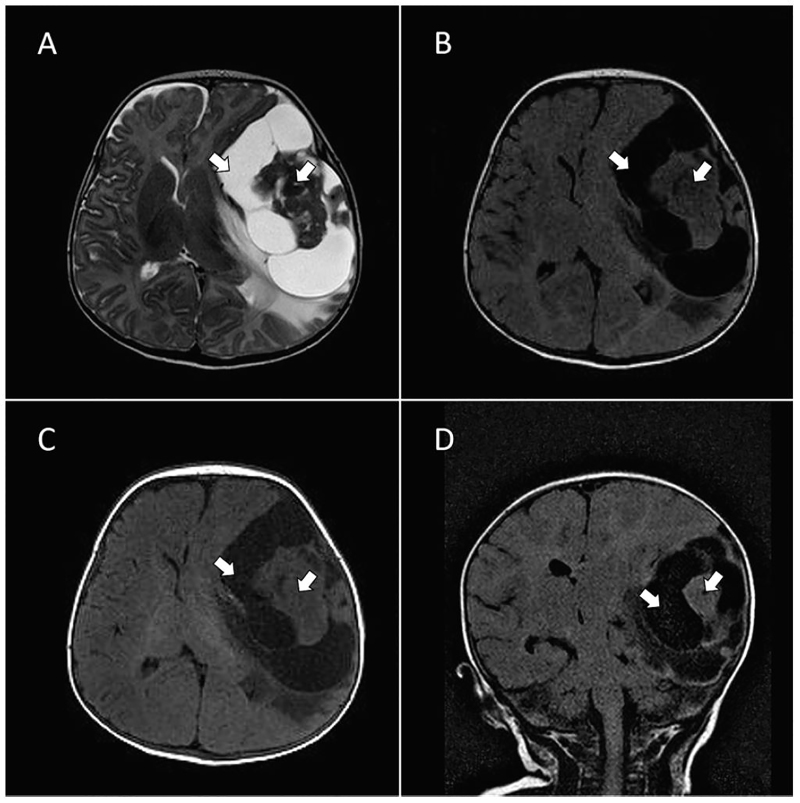
MRI revealing an 8.6cm × 7.9cm × 5.1cm semi-cysticsemi-solid mass (arrows) with epicenter in the left temporal region. T2-weighted axial view (A). T2-weighted with flair axial view (B). T1-weighted axial view (C). T2-weighed with flair coronal view (D). *MRI,* magnetic resonance imaging
